# Accuracy and reproducibility of T1rho mapping sequences

**DOI:** 10.1186/1532-429X-17-S1-P22

**Published:** 2015-02-03

**Authors:** Joyce Q Han, Yuchi Han, Walter R Witschey

**Affiliations:** 1 Cardiology, University of Pennsylvania, Philadelphia, PA, USA; 2 Radiology, University of Pennsylvania, Philadelphia, PA, USA

## Background

Previous studies have shown that T1rho-weighted imaging using long spin locking pulses enables high discrimination between infarct and myocardium without the need for exogenous contrast agents. To optimize imaging protocols, it's beneficial to understand the factors that influence the measurement. We measured the accuracy and reproducibility of spin echo-spin lock (SESL) T1rho-prepared balanced-steady-state free precession (bSSFP) sequences for myocardial relaxation time mapping. We subsequently performed *in vivo* studies and determined the average T1rho values in normal myocardium.

## Methods

**Figure 1:**
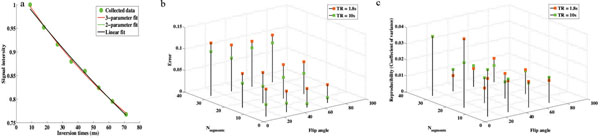
(a) The calculated T1rho values were 34.2, 55.0, and 58.6ms for 3-parameter, 2-parameter and linear fitting models respectively. (b) Accuracy of the 24 tested sequences assessed by the difference between each sequence and the gold standard weighted by reference T1rho values. Long TR and small number of segments produce more accurate results. (c) Reproducibility of the 24 tested sequences was assessed by the coefficient of variance. In contrary to results shown in the error maps, TR doesn't have a significant effect on reproducibility. Higher segments and higher flip angles are more reproducible.

A phantom containing 8 homogeneous H_2_O cylinders with MnCl_2_ concentrations of 0.01- 0.15 g/mL was used for the comparison of 24 T1rho mapping sequences with various repetition times (TR = 1.8-10 sec), flip angles (0-70 degrees), and numbers of segments (N_seg_ = 10-70). The scans were performed on a 1.5 T MRI (Avanto model, Siemens), and reference T1rho measurements were obtained by using gradient echo acquisition with TR = 10 sec, flip angle = 90 degrees, N_seg_ = 1. The overall scan time for this exam was approximately 1.5 hours. Three fitting methods were used to estimate T1rho recovering times,

linear model: log[S(t)] = -T_SL_/A + B;

2-parameter model: S(t) = B*exp(-T_SL_/A); and,

3-parameter model: S(t) = B*exp(-T_SL_/A) + C,

where t is the inversion time, A is the T1rho value, B is the initial magnetization M_0_ and C is the rms noise level (Figure 1a).

*In vivo* data were acquired on 10 healthy subjects on a 1.5 T scanner (Siemens). Two sequences were applied on each subject (TR = 1.8 sec, flip angle = 70 degrees, N_seg_ = 31).

## Results

For T1rho lower than 50 ms, we found that the 3-parameter model was the most accurate and reproducible. 2-parameter and linear models are preferred for higher T1rho values. As shown in Figure 1-a, the signal intensity decayed to the rms noise level (C, a positive nonzero value) and the 3-parameter fitting model is the only model that captured this behavior.

As shown in Figure 1-b and 1-c, substantial variations were observed in T1rho estimates measured and computed using preferred fitting models, from the 24 sequences. We observed that lower flip angle and higher N_seg_ increases T1rho underestimation, while higher flip angle and lower N_seg_ were more accurate and reproducible. SNR is related to the component of amplitude of the transverse magnetization which also varies with flip angle, there's a thus trade-off between T1rho measurement precision and accuracy. Although long TR and small N_seg_ allows the full relaxation of magnetization producing more accurate results, the resulting long scan time was not very practical in clinical settings.

**Figure 2:**
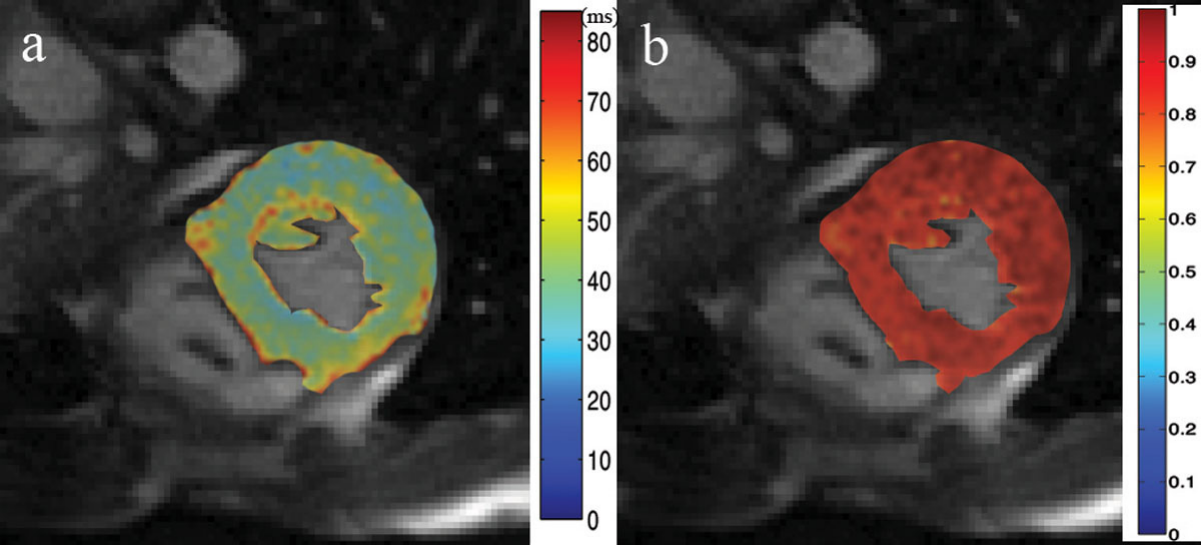
T1rho map of a normal subject, and the corresponding coefficient of determination map.

Figure 2 shows an example of *in vivo* T1rho and its error map obtained in a 24-year-old healthy male subjects.

## Conclusions

We described a number of adjustable factors in T1rho mapping. These factors depend on protocol adjustments, and can potentially influence the accuracy and reproducibility.

## Funding

None.

